# A novel OSA-related model of intermittent hypoxia in endothelial cells under flow reveals pronounced inflammatory pathway activation

**DOI:** 10.3389/fphys.2023.1108966

**Published:** 2023-04-13

**Authors:** Martin B. Müller, Clemens Stihl, Annika Schmid, Simon Hirschberger, Rea Mitsigiorgi, Martin Holzer, Martin Patscheider, Bernhard G. Weiss, Christoph Reichel, Max Hübner, Bernd Uhl

**Affiliations:** ^1^ Department of Anaesthesiology and Intensive Care Medicine, Research Unit Molecular Medicine, LMU University Hospital, Ludwig-Maximilians-University München (LMU), Munich, Germany; ^2^ Walter Brendel Center of Experimental Medicine (WBex), Ludwig-Maximilians-University München (LMU), Munich, Germany; ^3^ Department of Otorhinolaryngology, LMU University Hospital, Ludwig-Maximilians-University München (LMU), Munich, Germany

**Keywords:** obstructive sleep apnea, intermittent hypoxia (IH), endothelial cell dysfunction, cell culture models, culture under flow, inflammation, HIF, hypoxia inducible factor

## Abstract

Obstructive sleep apnea (OSA) is a common sleep-related breathing disorder characterized by recurrent episodes of upper airway obstruction and subsequent hypoxia. In patients with OSA, severity and number of these hypoxic events positively correlate with the extent of associated cardiovascular pathology. The molecular mechanisms underlying intermittent hypoxia (IH)-driven cardiovascular disease in OSA, however, remain poorly understood—partly due to the lack of adequate experimental models. Here, we present a novel experimental approach that utilizes primary human endothelial cells cultivated under shear stress. Oxygen partial pressure dynamics were adopted in our *in vitro* model according to the desaturation-reoxygenation patterns identified in polysomnographic data of severe OSA patients (*n* = 10, with 892 severe desaturations, SpO_2_<80%). Using western blot analysis, we detected a robust activation of the two major inflammatory pathways ERK and NF-κB in endothelial cells, whereas no HIF1α and HIF2α protein stabilization was observed. In line with these findings, mRNA and protein expression of the pro-inflammatory adhesion and signaling molecule ICAM-1 and the chemokine CCL2 were significantly increased. Hence, we established a novel *in vitro* model for deciphering OSA-elicited effects on the vascular endothelium. First data obtained in this model point to the endothelial activation of pro-inflammatory rather than hypoxia-associated pathways in OSA. Future studies in this model might contribute to the development of targeted strategies against OSA-induced, secondary cardiovascular disease.

## 1 Introduction

Obstructive sleep apnea (OSA) is a common sleep-related breathing disorder affecting almost one billion people worldwide (Benjafield et al., 2019; Lyons et al., 2020). It is characterized by recurrent upper airway obstructions with associated hypoxic episodes ultimately promoting cardiovascular diseases such as atherosclerosis, myocardial infarction, and stroke (Yeghiazarians et al., 2021). While studies have shown that the standard treatment for OSA patients by continuous positive airway pressure (CPAP) at least partially limit major adverse cerebrovascular events (Baguet et al., 2005; Muraja-Murro et al., 2013; Temirbekov et al., 2018; Javaheri et al., 2020), the treatment with CPAP failed to sufficiently prevent associated cardiovascular events in a large trial (McEvoy et al., 2016). Hence, OSA patients need novel therapeutic strategies to reduce their disease burden of secondary cardiovascular pathologies.

Interestingly, there is increasing evidence that the severity of the hypoxic events and the number of hypoxic events measured as oxygen desaturation index (ODI) correlate with OSA patient’s vascular pathology and mortality (Baguet et al., 2005; Muraja-Murro et al., 2013; Temirbekov et al., 2018). In particular, longer and deeper desaturations have been associated with increased OSA severity (Kulkas et al., 2013; Karhu et al., 2021). However, data on the underlying molecular mechanisms of how OSA-related intermittent hypoxia (IH) promotes vascular pathologies remain scarce.

Current *in vitro* models of IH regularly exhibit important limitations as they do not properly reflect the cycle characteristics and hypoxic oxygen concentrations of *in vivo* desaturations measured in patients with severe OSA (Morgan, 2009; Hunyor and Cook, 2018; Pavlacky and Polak, 2020). Moreover, current *in vitro* models used for studying endothelial pathophysiology in IH do not appreciate that endothelial cells exhibit their mature *in vivo* phenotype exclusively under flow conditions (Resnick and Gimbrone, 1995; Chistiakov et al., 2017).

To address these limitations, we analyzed the oxygen saturation time courses of single desaturation-reoxygenation cycles in the polysomnographic data from ten patients with severe OSA. On the basis of these data, we established a novel model of IH with shear stress-maturated primary endothelial cells cultured under flow, adequately mirroring the severe hypoxic event’s characteristics observed in OSA patients. Using this model, we explored the question which molecular mechanisms relevant for the development of cardiovascular diseases are activated in the endothelium by realistic OSA-related IH.

Here, we present, to our knowledge, the first *in vitro* model of IH investigating maturated endothelial cells under targetable shear stress and the first to be based on patient data resulting in a more pathophysiologically accurate model in comparison to presently established models. By employing our model, we found only moderate effects on classical hypoxic signaling, while our data revealed a markedly increased inflammatory endothelial activation after 4 h IH with 15 desaturations per hour to pathophysiological relevant pO_2_ levels. Hence, IH-induced inflammation might be a crucial driver and potential therapeutic target of chronic vascular diseases such as atherosclerosis, which are hallmarks of patients with severe OSA.

## 2 Materials and methods

### 2.1 Polysomnographic data

To depict the pathological characteristics of hypoxic cycles in OSA patients, we analyzed the oxygen saturation time course of single hypoxic events in polysomnographic data from ten patients with severe OSA (apnea hypopnea index, AHI >30 and with severe desaturations: peripheral oxygen saturation (SpO2) < 80%) diagnosed at the interdisciplinary sleep laboratory of the Department of Otorhinolaryngology, University Hospital, LMU Munich, Germany. Subjects gave written informed consent and the use of these data for research purpose was approved by the Institutional Ethics Committee of the Ludwig-Maximilian University Munich, Germany (Approval number 13–16). The in-laboratory polysomnogram recorded oximetry data which were processed by the automated software SleepWork™ 9 (Natus). The software-based calculation of scores and indices were reviewed by specialized technical assistants. We used the following definitions for oxygen desaturation index (ODI3%): average number of desaturations events with >3% decline in average oxygen saturation per hour of sleep as recommended by the American Academy of Sleep Medicine (Berry et al., 2012). For the detailed analysis of cycle characteristics, we manually reviewed each desaturation with SpO_2_ below 80%. These episodes were defined as severe desaturations and are associated with the presence of carotid plaque formation (Baguet et al., 2005; Piper et al., 2008). We measured the time from peak SpO_2_ until the nadir was reached (desaturation) and the time from nadir until oxygen saturation recovered and reached a subsequent peak (resaturation/reoxygenation).

### 2.2 Cell culture

Primary Human Umbilical Vein Endothelial Cells (HUVEC) were isolated from umbilical cords of healthy neonates directly after cesarean delivery at the Department of Gynecology and Obstetrics, University Hospital, LMU Munich, Germany. Written informed consent from the mother was obtained before donating umbilical cords in accordance with the Declaration of Helsinki. After collagenase A (Roche) treatment, HUVEC were detached from umbilical vein vascular wall and cultured in Endothelial Cell Basal Medium (ECGM; PromoCell) with Supplement Mix (PromoCell), supplemented with 10% FCS (Biochrom AG) and 1% Penicillin/Streptomycin (Pen/Strep, Gibco). A ViCell analyzer (Beckman Coulter, Fullerton, CA, United States) was used to evaluate the cell count and viability. For each of the independent experiments, we used non-pooled cells obtained from different donors at passage 2–4.

### 2.3 Flow experiments

µ-Slides I^0.2^ Luer (glass bottom channel, channel height 250 μm; ibidi^®^, Gräfelfing, Germany) were treated with 50 μg/mL Poly-L-Lysine for 20 min, 0.5% glutaraldehyde for 15 min, and 0.2% gelatin for 10 min followed by overnight incubation with ECGM culture medium to create surface coating for firm cell attachment. When HUVEC reached 80% confluency, cells were harvested and 120,000 of these were seeded into the channel slides. After 30 min of incubation cells attached to the bottom of the slide. Then, the channel slide was turned upside down and additional 120,000 cells were seeded on the opposing side of the channel slide. Cells were firmly attached after incubation for additional 3 h. Following a washing step, the slides were connected to the ibidi^®^ pump system’s fluidic unit with a red perfusion set (inner diameter 1.6 mm) and cultured under constant unidirectional flow overnight at a shear stress of 5 dyn/cm^2^. Thereafter, when HUVEC were completely aligned into the direction of flow the shear stress was increased to 15 dyn/cm^2^ to mimic flow conditions commonly observed in coronary or carotid arteries (Irace et al., 2012; Thondapu et al., 2017) for another 48 h.

### 2.4 Rapid cycling intermittent hypoxia model

**FIGURE 1:**
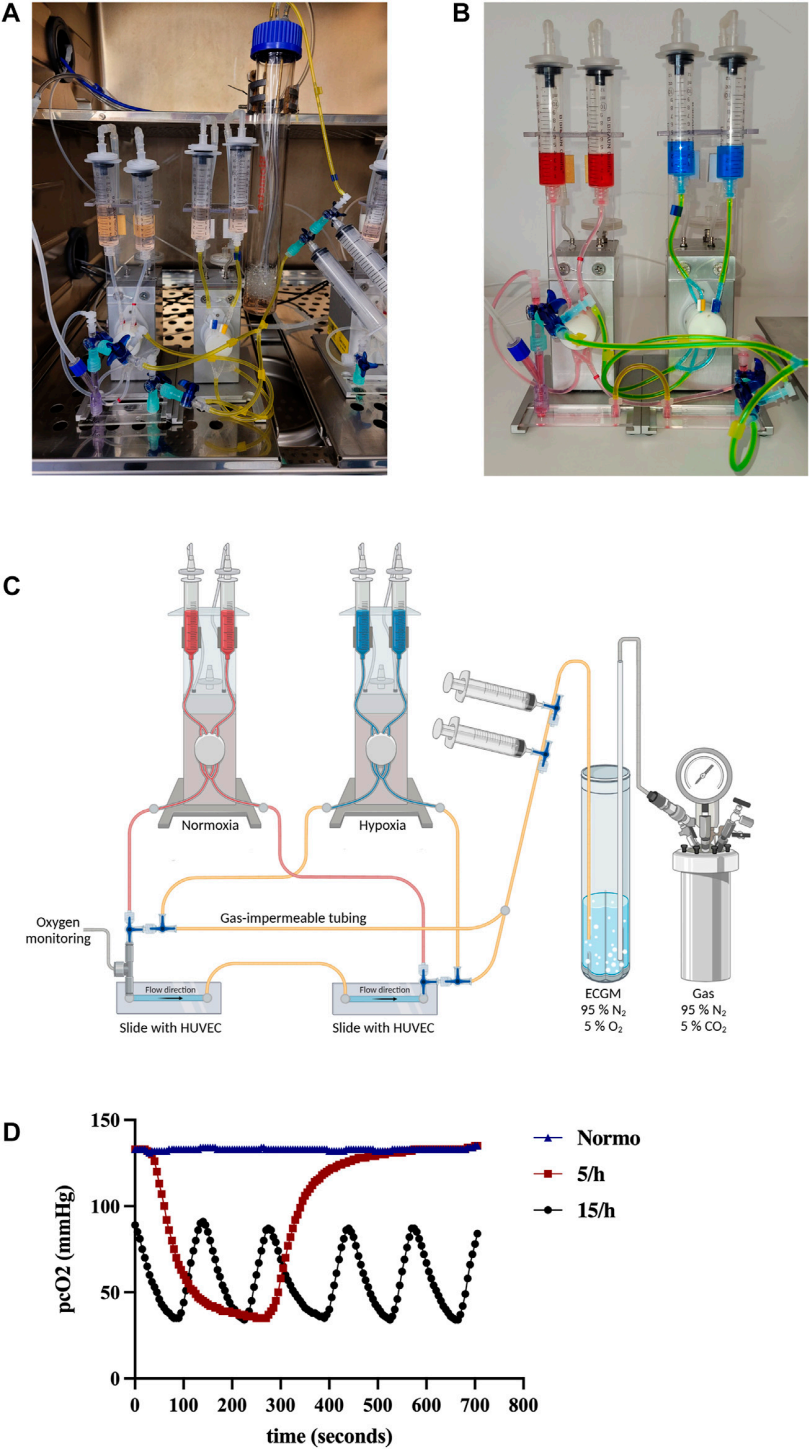
Introducing a novel model of intermittent hypoxia in endothelial cells under flow simulating hallmarks of OSA hypoxic pathophysiology. We set up a novel model reflecting the *in vivo* measured hypoxic desaturation characteristics (time course, frequency and target oxygen concentration). **(A)** Experimental overview of the novel model under cell culture conditions in the incubator **(A)**, in detail **(B)** and as schematic illustration **(C)**: Desaturated medium is illustrated by blue color, normal medium by red color. The medium is constantly degassed in the bioreactor with 95% N_2_ and 5% CO_2_ and intermittently loaded into the “hypoxic” fluidic unit indicated by gas impermeable yellow tubing before each hypoxic cycle. Switching from normal to the hypoxic medium directed by different perfusion systems can be achieved by rotating the stopcock’s position (Supplementary Video S1). The endothelial cells can be perfused with the degassed medium until the real-time oxygen measurement confirms the targeted oxygen partial pressure (Supplementary Video S2). Detailed partial pressure of oxygen on cellular level (pcO_2_) over time measured by LiCox^®^ probe **(D)**. Shown are 5 subsequent hypoxia/reoxygenation cycles in the 15/h group and 1 cycle in the 5/h group, while the normoxia group stayed on atmospheric oxygen partial pressure.

For preparation of our novel rapid cycling IH model, 30 mL of ECGM culture medium was constantly degassed with 95% N_2_ and 5% CO_2_ in a glass bioreactor (Biometra) to create hypoxic medium. The whole setup is placed in a humidified incubator with 37°C and 5% CO_2_ (Figure 1A). Using three-way stopcocks, gas impermeable tubes (Tygon^®^ F-4040), and syringes, the bioreactor was connected with the reservoir syringes (10 mL, Braun) of a customized perfusion set, enabling the operator to pump the degassed medium into the flow setup (Figure 1B and schematic overview, Figure 1C, created with BioRender.com). A Y-connector was placed at the entrance of the slide serving as insertion point for a Licox^®^ probe (Brain Tissue Oxygen Monitoring system, Integra^®^), which is usually used to quantify brain tissue oxygenation as part of intensive care unit neuromonitoring, in order to measure real time partial pressure of O_2_ on a cellular level (pcO_2_). Before each hypoxic cycle, the degassed medium was pumped into the gas impermeable tube system of one fluidic unit. The fluidic unit with hypoxic medium was connected to a gas cylinder filled with 95% N_2_ and 5% CO_2_ using a precision pressure regulator (R216-02E, G¼, 0.01–0.6 bar, AirCom^®^) to avoid reoxygenation of the medium. Directly before the entrance of the glass slide, the three-way stopcocks can direct the hypoxic medium into the slide (Supplementary Video S1). Oxygen partial pressure is visible at the Licox^®^ monitor (Supplementary Video S2). Switching from normal to the hypoxic pipe system can be achieved by rotating the stopcock’s position. The end of each cycle can be induced by switching the stopcock towards the perfusion system of a second fluidic unit with normal non-hypoxic medium. The correct time to cease the hypoxic cycle and to induce reoxygenation is determined by the real-time measurement of the oxygen partial pressure using the Licox^®^ probe. We perfused the hypoxic medium either 5 or 15 times per hour through the slide in order to mimic 5 or 15 hypoxic events per hour referred as ODI (severe) in patients. After 15 cycles for each hour had been executed in the 15/h group, the pcO_2_ slowly increased to end up at atmospheric levels (130–140 mmHg) until the next consecutive 15 cycles started (Supplementary Figure S1). In the 5/h group, the pcO2 also rose to atmospheric levels between the single (longer) cycles (130–140 mmHg). As a negative control, we intermittently perfused non-hypoxic medium with an atmospheric partial pressure of oxygen (130–140 mmHg) through the slide 15 times per hour. After 4 h of IH, cells were washed and detached by incubation with prewarmed Accutase (Sigma Aldrich) at 37°C and 5% CO_2_. Then cells were carefully flushed out of the slide using 1 mL syringes filled with ECGM with 10% FCS to stop the Accutase reaction. Cells were pelleted, washed and either lysed for RNA and protein analysis or stained for flow cytometry.

### 2.5 Hypoxia detection

To confirm early and low-dose hypoxia in our endothelial cells, we stained the channel slides with attached HUVEC prior to the hypoxic cycles with Image-iT™ green hypoxia reagent (Invitrogen™) for 20 min according to the manufacturer’s protocol. This fluorescent molecular probe is sensitive to varying concentrations of oxygen, already reacts at O_2_ concentrations starting from 5% and due to its irreversible fluorescence is capable of indicating the cumulative hypoxic burden of repetitive hypoxia/normoxia cycles (Zhang et al., 2021). After 4 h of IH, the slides were disconnected from perfusion systems and were further investigated under the LEICA-TCS SP5 confocal microscope. Images were acquired using the Leica application suite AF software, version 2.7. After microscopy image acquisition, cells were enzymatically detached for a sensitive flow cytometry analysis of the Image-iT™ green hypoxia reagent fluorescence intensity on a single cell level.

### 2.6 Flow cytometry

Flow cytometric analysis was preformed using a FACS Canto II flow cytometer (BD Biosciences, Franklin Lakes, NJ, United States). For staining of cell surface expression of ICAM-1, unspecific binding was blocked with 5% FCS in PBS for 5 min at room temperature (RT). Cells were stained with fluorophore-conjugated antibodies directed against ICAM-1/CD54 (1:25 PE mouse anti human CD54, BD Pharmingen). Data were analysed using FlowJo software, version 10 (FlowJo™, BD).

### 2.7 ELISA

Protein levels of secreted ICAM-1 were quantified in cell culture medium by an enzyme-linked immunosorbent assay (RayBio^®^ Human sICAM-1 ELISA Kit, RayBiotech). Assays were performed according to the manufacturer’s protocol. Absorbance was measured on a Filtermax F3 and values evaluated by using a plate-specific standard curve.

### 2.8 Quantitative real-time PCR

RNA was isolated from HUVEC with a miRNeasy Mini kit (Qiagen) according to the manufacturer’s protocol. RNA concentration and quality was measured using a NanoDrop 2000 spectrophotometer (ThermoFisher). cDNA was synthesized from RNA using Oligo-dT Primers, Random Hexamers (Qiagen), dNTPs, RNAse OUT, and Superscript^®^ III Reverse Transcriptase (Invitrogen). Quantitative real-time PCR (qRT-PCR) was performed using specially designed primers (Probe Finder Software: Roche; primer: Metabion, Martinsried) and respective UPL Probes (Roche) on a Light Cycler 480 instrument (Roche) with 10 ng of cDNA/well as previously described (Muller et al., 2022). Primer sequences: CCL2, for: AGT​CTC​TGC​CGC​CCT​TCT, rev: GTG​ACT​GGG​GCA​TTG​ATT​G; ICAM-1, for: CCT​TCC​TCA​CCG​TGT​ACT​GG, rev: AGC​GTA​GGG​TAA​GGT​TCT​GC; GAPDH, for: AGC​CAC​ATC​GCT​CAG​ACA​C, rev: GCC​CAA​TAC​GAC​CAA​ATC​C; TBP, for: GAA​CAT​CAT​GGA​TCA​GAA​CAA​CA, rev: ATA​GGG​ATT​CCG​GGA​GTC​AT) All analyses were performed in duplicates. Glyceraldehyde 3-phosphate dehydrogenase (GAPDH) and TATA Box Binding Protein (TBP) were used as reference genes. Quantification cycle values were calculated by the “second derivative maximum” method computed by the LightCycler^®^ software.

### 2.9 SDS-PAGE

HUVEC were lysed in cell lysis buffer containing 1% protease and phosphatase inhibitors (all Cell Signaling Technologies). Protein concentrations were determined with BCA assays (ThermoFisher), SDS-PAGE was performed and protein was electroblotted on polyvinylidene difluoride (PVDF) membranes using the Trans-Blot Turbo Transfer System (Biorad). Non-specific binding was blocked by incubating the membranes with 5% non-fat milk for 1 h. Primary antibodies for HIF1α (#36169), HIF2α (#71565), p-IkB (#2859; all Cell Signaling Technologies), and p-ERK (sc-7383 Santa Cruz Biotec) were diluted in TBST with 5% non-fat milk. β-Actin (#4970; Cell Signaling Technologies) served as the loading control. Immunoreactive bands were visualized following incubation with peroxidase-conjugated secondary antibodies (Anti-rabbit IgG, #7074; Anti-mouse IgG, #7076; Cell Signaling Technologies) and subsequent incubation with the ECL western blot detection substrate (BioRad) using a CCD imager. As internal positive controls for HIF stabilization, HUVEC treated with 5% and 1% constant hypoxia or 1 mM DMOG for 4 h were used. As positive control for inflammatory activation, HUVEC were stimulated with 25 ng/mL TNF for 24 h.

### 2.10 Statistics

Values are presented as means ± standard error of the mean (SEM) if not stated otherwise. N refers to the number of independent experiments with cells obtained from different HUVEC donors. The measured values in the three groups (negative control = *normo*; 5 hypoxic events per hours = *5/h*; 15 hypoxic events per hour = *15/h*) were tested for normal distribution with Shapiro Wilk test. For normally distributed data, the *p*-values were calculated using repeated-measures one-way ANOVA with the Geisser-Greenhouse correction and with Turkey’s multiple comparison test to correct for multiple comparisons. For non-normally distributed data, we used a Friedman test with Dunn’s multiple comparison test. Statistical analyses were performed using GraphPad Prism version 9.3.0. *p*-values lower than 0.05 were considered statistically significant (*p < 0.05; **p < 0.01; ***p < 0.001).

## 3 Results

### 3.1 Polysomnographic analysis of patients diagnosed with severe OSA

In order to investigate the typical oxygen saturation time course of representative severe desaturation-reoxygenation cycles in patients with severe OSA, we analyzed polysomnographic data of ten patients with an apnea hypopnea index (AHI) > 30 with regard to hypoxic events with a SpO_2_ < 80% (Table 1). The patients were all male with an average age of 43.8 (±13.2, all SD) years and a body mass index (BMI) of 32.7 (±3.7) kg/m^2^. The analysis revealed an AHI and ODI3% of 65.3 (±13.5) and 65.5 (±15.5). While the patients exhibited 14.6 (±14.9) severe desaturation per hour, we measured a mean SpO_2_ nadir of 66.6% (±7.7). The average SpO_2_ measured while the patients were awake was 92.7% (±1.2).

**TABLE 1:** OSA patient's characteristics and polysomnographic analysis.

Patient	Age (years)	BMI (kg/m2)	AHI	ODI	ODI (severe)	Time to nadir (seconds)	Time to peak (seconds)	# desat. SpO2 <80%	SpO2 nadir, (%)	Average SpO2 awake
1	49	36.0	87.8	85.1	8.03	38.0	11.3	40	68.7	92.3
2	49	23.5	64.1	45.9	4.49	47.0	14.2	27	64.8	94.2
3	31	29.1	62.4	71.6	13.2	47.0	12.6	87	75.5	92.0
4	21	30.0	74.9	69.6	42.9	42.1	9.4	252	50.0	94.8
5	37	26.9	41.3	39.6	4.0	44.0	13.3	24	69.6	93.0
6	38	30.2	70.0	70.0	4.7	43.7	33.2	20	76.3	92.9
7	52	38.0	70.9	85.6	36.1	41.1	9.7	148	57.6	90.7
8	73	28.4	50.1	62.6	6.1	44.9	12.1	41	64.8	92.3
9	45	28.4	64.9	88.7	0.8	49.0	13.6	6	73.0	91.9
10	43	34.9	60.1	56.0	30.0	37.2	11.4	247	65.6	89.4
COHORT	43.8 (13.2)	32.7 (3.7)	65.3 (13.5)	65.5 (15.5)	14.6 (14.9)	43.6 (3.8)	14.0 (6.9)	892 (94.0)	66.6 (7.7)	92.7 (1.2)

Analysis of ten patients with severe OSA (AHI >30 and severe desaturations <80% SpO_2_): BMI, body mass index; AHI, apnea hypopnea index; ODI, oxygen desaturation index; ODI (severe), includes only severe desaturations (SpO_2_<80%); Time to nadir, defined as desaturation is the time from peak to nadir SpO_2_ in seconds; Time to peak, defined as resaturation/reoxygenation is the time from nadir SpO_2_ to peak in seconds; # desat. SpO_2_ <80%, number of severe desaturations <80% SpO2; SpO2 nadir, is defined as the lowest measured SpO_2_ during an apnea. The data presented in the overall cohort are means (± standard deviation).

In total, we analyzed 892 severe desaturations. The mean time from oxygen saturation peak to the oxygen saturation nadir (desaturation) was 43.6 (±3.8) seconds, whereas the mean time from oxygen saturation nadir to peak oxygen saturation (resaturation) was 14.0 (±6.9) seconds, thus taking one-third of the time as the desaturation episode.

### 3.2 Translation into a cell culture model of intermittent hypoxia

There is a lack of IH *in vitro* models adequately simulating desaturation patterns relevant in patients with severe OSA. Therefore, we translated our polysomnographic data into a rapid cycling IH cell culture model with primary shear stress-maturated endothelial cells in order to investigate vascular pathophysiologic processes occurring during OSA in a more realistic setup.

The here presented cell culture model is driven by air pressured flow pumps (experimental setup is detailed in Figure 1A–C). The aim was to depict OSA-related vascular pathologies occurring in larger vessels such as coronary and carotid arteries. Thus, 15 dyn/cm^2^ was selected as the target shear stress, representing the mean shear stress present in the aforementioned vessels. Endothelial cells in these arteries are exposed to the arterial partial oxygen pressure. Under homeostatic conditions, it ranges from 75 to 100 mmHg (Collins et al., 2015) and drops during the hypoxic events in OSA patients to the nadir of arterial partial pressure, which correlates with peripheral oxygen saturation (Severinghaus, 1979; Madan, 2017). To simulate these conditions, we extrapolated the *in vivo* measured peripheral oxygen saturation nadirs of the OSA patients (SpO_2_ 66.6%) into a target oxygen partial pressure (35 mmHg) measured on cellular level (pcO_2_) in our *in vitro* model by using the oxyhemoglobin dissociation curve (Severinghaus, 1979) (Table 1). Since the investigated patients had in average 15 severe desaturation per hour, we studied three groups with gradual increase in hypoxic events (negative control = *normo*; 5 hypoxic events per hours = *5/h*; 15 hypoxic events per hour = *15/h*).

Analysis of the model’s hypoxic cycle characteristics was performed in the 15/h group, since the desaturation/reoxygenation cycles of the 5/h group took substantially longer. The mean desaturation time was 78.00 (±4.95) seconds and the resaturation time was 46.00 (±7.16) seconds (Table 2; Figure 1D). Hence, our model did not completely reach the short cycling durations measured in our selected patient cohort with severe OSA experiencing deep and prolonged desaturations; however, at least the 15/h group decently simulated the fundamental cycle characteristics with shorter resaturation than desaturation durations (Table 2; Figure 1D). The real-time measured partial pressure of oxygen on cellular level (pcO_2_) using a highly sensitive electrode was 34.80 (±1.05) mmHg, while the peak pcO_2_ was 84.6 (±2.39) mmHg (Table 2; Supplementary Figure S1), providing proof that the target partial pressure of oxygen relevant for OSA patients was reliably and reproducibly reached in this model. Thus, we can conclude that this novel experimental approach decently mimics certain *in vivo* measured hallmarks of OSA-related severe desaturations (targeted oxygen concentration, shape of the de- and resaturation cycles, frequency of desaturations), while some hallmarks could be simulated adequately, but not perfectly (duration of desaturation and resaturation).

**TABLE 2:** Hypoxic cycle characteristics reached in the model.

Model	Time to nadir (seconds)	Time to peak (seconds)	Nadir pcO_2_ (mmHg)	Peak pcO_2_ (mmHg)
*n* = 1	75	47	35.80	85.60
*n* = 2	72	40	36.20	85.50
*n* = 3	80	41	34.40	87.50
*n* = 4	85	58	33.60	81.00
*n* = 5	78	44	34.00	83.40
Mean (±SD)	78 (4.95)	46 (7.16)	34.80 (1.05)	84.60 (2.39)

Analysis of the model’s hypoxic cycle characteristics. Time to nadir, defined as desaturation, is the time from beginning of the desaturation (pcO_2_, partial pressure of oxygen on cellular level, <80 mmHg) to nadir pcO_2_ in seconds; Time to peak, defined as resaturation/reoxygenation is the time from nadir back to 75 mmHg pcO_2_ in seconds. The presented data are means of 5 subsequent cycles per experiment (± standard deviation).

### 3.3 Intermittent hypoxia leads to weak canonical hypoxic signaling

**FIGURE 2:**
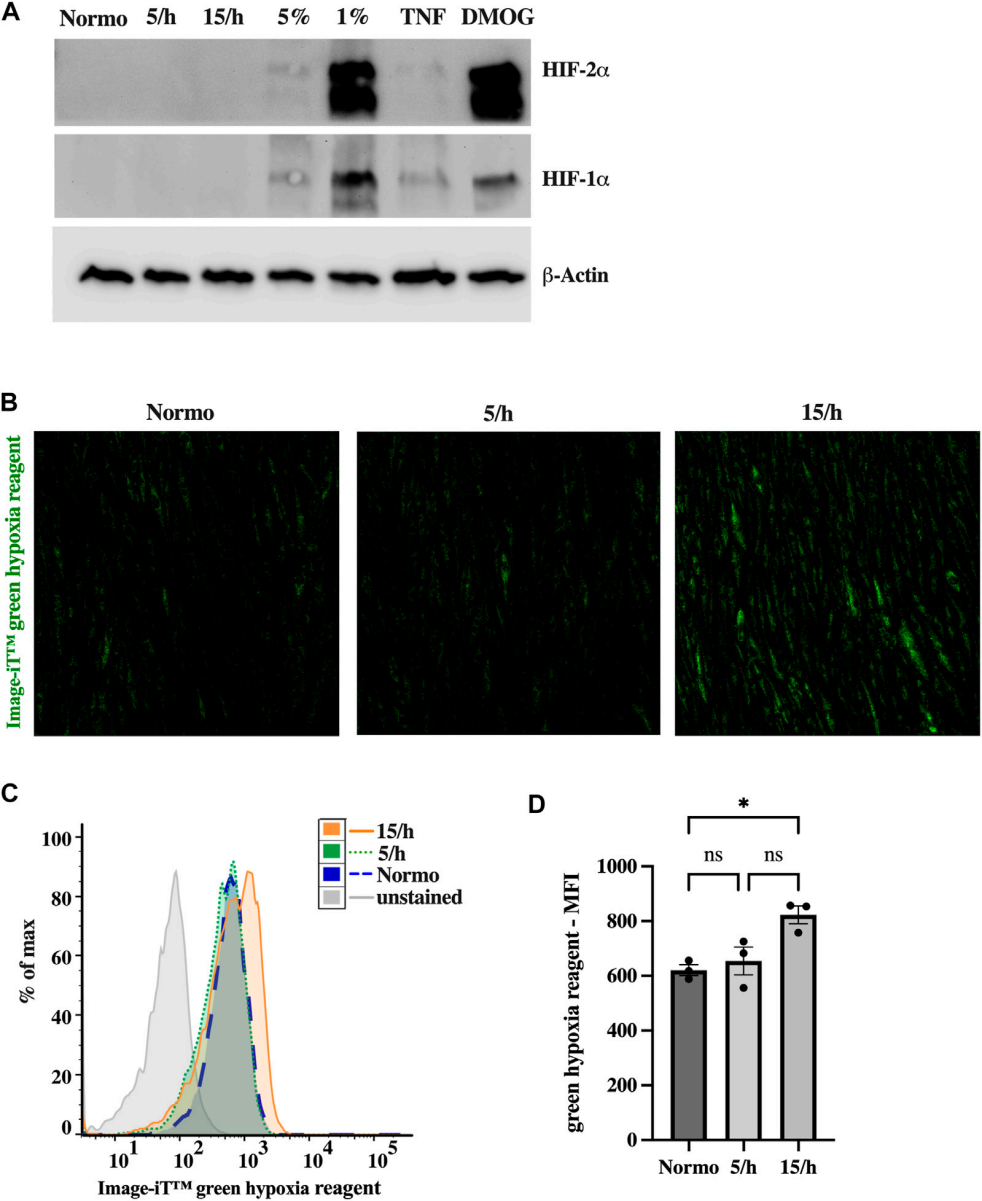
Intermittent low grade hypoxia (pcO_2_ 35 mmHg 15 cycles per hour, for 4 h) does not trigger HIF1α and HIF2α stabilization, but activates sensitive hypoxic molecular probe. After 48 h of 15 dyn/cm^2^ shear stress endothelial cells were treated with 5 or 15 cycles per hour of intermittent hypoxia for 4 h. Western Blots of HIF1α and HIF2α **(A)** did not show any expression in endothelial cell lysates after intermittent hypoxia. In contrast, 5%, and in particular 1% of constant hypoxia or the treatment with Dimethyloxalylglycine (DMOG, 1 mM), an inhibitor for HIF prolylhydroxylases, for 4 h did stabilize HIF1α and HIF2α. Inflammatory activation (TNF 25 ng/mL) resulted in slight HIF expression. However, using a sensitive marker of low dose hypoxia (Image-iT™ green hypoxia reagent), we detected a significant increase in staining following 15 hypoxic cycles per hour in confocal microscopy **(B)**. Upon completion of confocal images, HUVECs were detached and subjected to detailed fluorescence analysis on a single cell level by flow cytometry. **(C)** shows a representative histogram of one experiment depicting the mean fluorescence intensity (MFI) of Image-iT™ green hypoxia reagent in all experimental groups. The data of three independent experiments are summarized in the bar graph of **(D)**. Shown are means ± SEM. *n* = 3–6, *p < 0.05.

As a next step, we investigated the cellular hypoxic burden induced by the repetitive hypoxic cycles. The analysis of hypoxic signaling using western blots did not show HIF1α and HIF2α stabilization after 4 hours of IH with 15 hypoxic events per hour and a target pcO_2_ of 35 mmHg (Figure 2A). In contrast, constant hypoxia with 5% and particularly 1% of oxygen, or stimulation with DMOG for 4 h clearly induced HIF stabilization. To confirm these results, we additionally studied hypoxia using Image-iT™ green hypoxia reagent. In line with the absence of HIF stabilization, we could detect only slight hypoxia in the 15/h group using confocal microscopy (Figure 2B) and flow cytometry (Figure 2C, D). However, compared to the non-hypoxic control, the 15/h group showed a significantly increased fluorescence signal, while the group with 5/h did reveal a non-significant increase, indicating a lager cumulative hypoxic burden in the group with 15/h compared to 5/h.

In summary, 60 cycles of short hypoxic events during a 4 h period was not sufficient sustained hypoxia to trigger stable HIF1α and HIF2α expression, but rather induces early weak hypoxic signaling potentially preceding HIF protein accumulation.

### 3.4 Intermittent hypoxia leads to a gradual increase of inflammatory signaling depending on the number of hypoxic cycles

OSA patients often present clinical signs of chronic systemic low-grade inflammatory activation (Kheirandish-Gozal and Gozal, 2019). To evaluate the hypothesis, whether IH triggers an inflammatory activation comparable to the one observed in patients, we investigated the effects of IH on the expression of key molecules of the inflammatory reaction in endothelial cells: The adhesion and signaling molecule Intercellular Adhesion Molecule 1 (ICAM-1) and the chemotactic cytokine CC-chemokine ligand-2 (CCL2).

**FIGURE 3:**
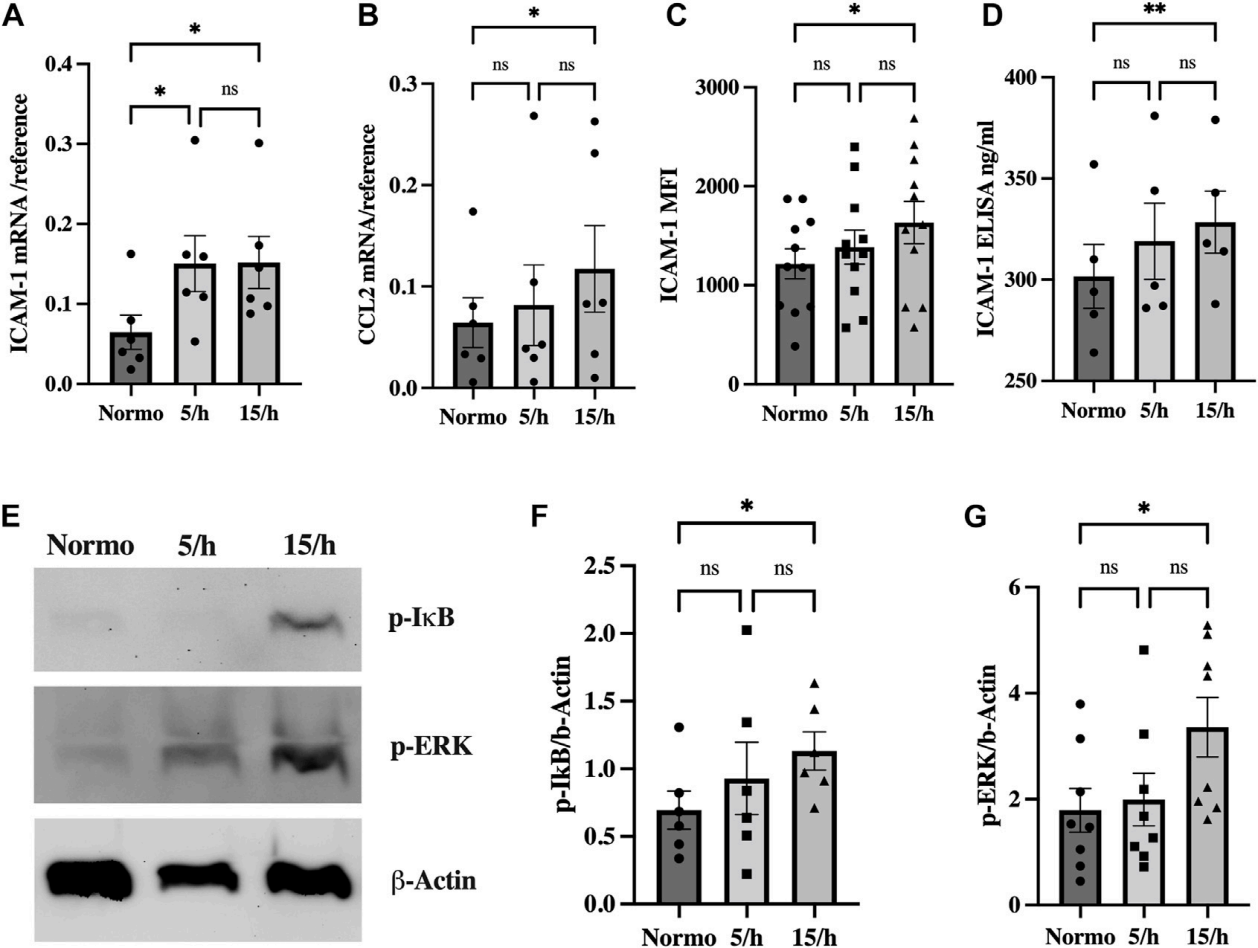
Intermittent hypoxia induces inflammatory signaling driven by NF-κB and ERK activation to upregulate the adhesion and signaling molecule ICAM-1 and the chemokine CCL2. We detected an increased mRNA expression of the adhesion molecule ICAM-1 **(A)** and the chemokine CCL2 **(B)** in qRT-PCR in the group with 15 hypoxic events per hour compared to the normoxic control. Accordingly, ICAM-1 surface expression detected in flow cytometry **(C)** and cleaved ICAM-1 concentration in the cell culture media measured by ELISA **(D)** were significantly increased. Western blot analysis showed significantly elevated p-IkB and p-ERK **(E)** expression as surrogate for NF-κB and ERK activation already in the 5/h group, finally reaching significant levels in the group with 15 cycles per hour **(F, G)**. Shown are means ± SEM. *N* = 6–10, *p = 0.05, **p = 0.01.

We indeed found a significant endothelial mRNA induction of ICAM-1 in the 5/h and 15/h groups and of the chemokine CCL2 in the 15/h group as compared to the control group (Figures 3A, B). In line with this data, ICAM-1 protein expression was also increased on the endothelial cell surface and in the perfused cell culture medium of the 15/h hypoxic group as compared to the control group perfused with normoxic medium (Figures 3C, D). As potential inflammatory activators of these inflammatory molecules on a transcriptional level, we detected a significantly enhanced expression of phosphorylated ERK and IkB protein in western blots in the group with 15/h hypoxic events (Figures 3E–G). Our data indicate a substantial activation of the proinflammatory pathways ERK and NF-kB, which have been related to the cellular response on IH.

In conclusion, exposing endothelial cells under flow to 60 short hypoxic events resulted in slight hypoxic signaling, however, inflammatory pathways were markedly activated leading to an increased expression and/or secretion of pathophysiologically relevant inflammatory molecules.

## 4 Discussion

Obstructive sleep apnea (OSA) is a frequent sleep-related breathing disorder characterized by recurrent upper airway obstructions with associated hypoxic events. Even though OSA patients considerably suffer from immediate effects such as sleep fragmentation and fatigue, particularly the secondary cardiovascular pathologies including arterial hypertension, coronary artery disease, and myocardial infarction pose a substantial disease and healthcare burden (Yeghiazarians et al., 2021). Since a recent large trial indicated that standard treatment by CPAP is not able to sufficiently prevent OSA associated cardiovascular events (McEvoy et al., 2016), it appears to be crucial to identify novel therapeutic strategies for OSA patients to reduce the disease burden of secondary cardiovascular pathologies. Therefore, it is mandatory to deepen the knowledge on the underlying molecular mechanisms by which IH triggers chronic vascular pathologies.

Noteworthy, present standard *in vitro* models used to study molecular effects of IH often ignore several important hallmarks of OSA vascular pathology. Thus, establishing new pathophysiologically adequate models is a prerequisite to be able to characterize the actual underlying mechanisms required to identify novel therapeutic targets (Morgan, 2009; Hunyor and Cook, 2018; Pavlacky and Polak, 2020). Most cell culture models of IH published to date still use the conventional approach of changing the concentration of oxygen in the atmosphere surrounding the culture medium (Hoffmann et al., 2013; Huang et al., 2013; Chuang et al., 2014; Sharma et al., 2018; Wohlrab et al., 2018; Chuang et al., 2019; Sun et al., 2019; Hao et al., 2021; Regev et al., 2022). However, the slow oxygen gas-liquid diffusion leads to longer incubation times under hypoxia (up to 30 min), which limits the number of hypoxic cycles per hour (1–8/h) and prevents reaching pathophysiologically relevant oxygen concentration for the studied cell types. Hence, IH reaching only inadequately high oxygen concentrations assumably does not significantly promote OSA related pathways while unrealistically low oxygen concentrations might overactivate these signaling pathways or activate different pathways. In conclusion, most current *in vitro* models addressing IH exhibit important limitations by not properly implicating the cycle characteristics and hypoxic oxygen concentrations of *in vivo* desaturations measured in patients with severe OSA (Morgan, 2009; Hunyor and Cook, 2018; Mateika, 2019).

Interestingly, recently published *in vitro* models employing gas-permeable membranes, on which the cells are seeded, are technically feasible to exhibit hypoxic cycling rates and oxygen concentrations resembling *in vivo* conditions in patients with severe OSA (Campillo et al., 2017; Minoves et al., 2017). However, the endothelial cells in these models have been cultivated under static conditions thereby ignoring the fact that endothelial cells exhibit their *in vivo* shear stress-maturated phenotype exclusively under flow conditions (Resnick and Gimbrone, 1995; Chien, 2007; Chistiakov et al., 2017).

In our model, we were able to overcome the aforementioned limitations by alternately perfusing primary human endothelial cells with hypoxic or normoxic medium using a commercially available flow pump system. On the one hand, this method allowed to culture endothelial cells under targetable unidirectional shear stress for several days, simulating shear conditions in the blood vessel of interest. On the other hand, by using previously deoxygenated medium in this model, it is possible to instantly expose the endothelial cells under investigation to either hypoxic or normoxic conditions thereby enabling rapid desaturation and reoxygenation cycles directly on a cellular level. To finally establish a translational, pathophysiologically relevant cell culture model of IH, we adopted the polysomnographic parameters from ten severe OSA patients regarding the oxygen saturation cycle characteristics of severe desaturations (SpO2<80%), since deeper and longer hypoxic events are associated with an increased risk of secondary pathologies (Kulkas et al., 2013; Karhu et al., 2021). We detected a non-sigmoidal cycle slope with faster reoxygenation as compared to desaturation, which was already described as a typical characteristic of OSA-related desaturations (Lim et al., 2015). By simulating the *in vivo* measured temporal patterns, oxygen concentrations and frequency of hypoxic events, this model decently mimics most of the characteristics of IH as it was observed in our cohort of OSA patients with severe desaturations.

Regarding the effects of IH on endothelial cells, a sensitive hypoxia probe did only detect a slight increase in hypoxia although applied to the highest hypoxic burden in our model (15/h). In contrast to findings from other IH models, we did not see significant HIF1α and HIF2α accumulation in endothelial cells after in total 60 short hypoxic cycles (15/h) (Yuan et al., 2008; Regev et al., 2022). However, our results are in line with a previous study stating that IH (4 cycles per hour) to physiological oxygenation levels is not sufficient to induce HIF1α signaling (Ryan et al., 2005). Further characterizing the differences between IH and permanent hypoxia, we could demonstrate that permanent hypoxia using 5% of oxygen, which is comparable to the target oxygen partial pressure in our IH model, stabilized HIF1α and HIF2α slightly. In contrast, a 4 hour incubation with permanent hypoxia of 1% of oxygen clearly induced HIF stabilization. Our data are in line with different publications showing that various other cell types similarly require lower oxygen concentrations or prolonged exposure in order to mediate HIF1α and HIF2α accumulation in a time-dependent manner (Bracken et al., 2006; Bartoszewski et al., 2019; Jaskiewicz et al., 2022). From a translational perspective, it needs to be considered that coronary artery endothelial cells are not exposed to such low oxygen concentrations during OSA desaturations further underlining the need for more physiological OSA models.

Rayn and others have shown that endothelial cells exposed to IH rather show an inflammatory activation than classical HIF hypoxic signaling (Ryan et al., 2005). Corroborating these results, endothelial cells exposed to IH in our model similarly exhibited an inflammatory phenotype even when experiencing only slight oxygen desaturations. ICAM-1 is a key adhesion and signaling molecule in the inflammatory response promoting leukocyte-endothelium-interactions. Increased soluble ICAM-1 is commonly seen in the blood of OSA patients after being shedded from the endothelial cell surface and has already been shown to correlate with OSA disease severity (Ursavas et al., 2007; Haranczyk et al., 2022; Peracaula et al., 2022). Employing our model, we found an increased mRNA and surface protein expression of ICAM-1, as well as elevated secreted protein present in the media of the 15/h group as compared to the normoxia control group, thereby underlining its translational character. Moreover, 15 hypoxic events per hour increased the endothelial mRNA expression of CCL2, an essential chemotactic cytokine in the inflammatory response. These results are in accordance with a study of Polotsky et al. showing that IH induced proinflammatory cytokine production in aortic endothelial cells (Polotsky et al., 2010). Thus, IH-triggered endothelial inflammation and subsequent leukocyte trafficking might significantly promote atherosclerosis development (Sun et al., 2019).

Towards a mechanistic understanding, it has already been shown that ICAM-1 and CCL2 are transcriptionally activated by NF-kB and ERK. In line with our previous results, both pathways are significantly activated in our, but also other IH models investigating endothelial cells (Ryan et al., 2005; Dyugovskaya et al., 2012; Han et al., 2013; Makarenko et al., 2014). This indicates that IH activates non-canonical hypoxic signaling independent of HIF stabilization, potentially by a mechanism in which cells exposed to repetitive cellular oxygen tension might induce inflammation comparable to reperfusion injury. For example, increased ROS production as a result to mitochondrial and metabolic alterations is known to activate classical inflammatory transcription factors during repetitive episodes of hypoxia (Schonenberger and Kovacs, 2015).

The here presented model does also face limitations: We did not investigate long-term effects, thereby, we cannot exclude that HIF signaling is induced by our IH model when cycles are performed for a longer period than 4 h. Additionally, since we did not completely reach the fast desaturation and resaturation periods as measured in our cohort and the oxygen characteristics do not perfectly match between the different investigated groups in terms of the reached peak normoxic pcO_2_ during the cycles, we cannot exclude direct effects of these longer exposure periods on hypoxic and peak hyperoxic oxygen concentrations on HIF stabilization and inflammatory signaling. Another limitation is that the model is not automated and needs human assistance for its use. In this context, a computer regulated version would be favorable to avoid potential errors and increase the duration of the investigated periods. Finally, the extrapolation of partial pressure of oxygen values from an oxyhemoglobin dissociation curve does not adequately implicate important conditions present in the tissues of OSA patients, e.g., intermittent hypercapnia, potentially shifting the dissociation curve and ultimately affecting endothelial cells. Subsequent *in vivo* animal studies might be suitable to appropriately address these limitations.

In summary, to our knowledge, this novel model allows to investigate the molecular changes in shear stress-maturated endothelial cells under OSA-analogous IH for the first time. Employing our model, we could identify an inflammatory activation in response to IH, whereas this stimulus was not sufficient to trigger canonical hypoxic signaling. Thus, we provide new insights into inflammatory endothelial cell activation triggered by IH. Improved understanding of the underlying mechanisms of IH-mediated vascular pathologies in OSA patients, in turn, might pave the way for new treatment strategies.

## Data Availability

The raw data supporting the conclusion of this article will be made available by the authors, without undue reservation.
