# Supplementary material for: A novel OSA-related model of intermittent hypoxia in endothelial cells under flow reveals pronounced inflammatory pathway activation
Source: Front Physiol. 2023 Apr 13;14:1108966. doi: 10.3389/fphys.2023.1108966 (PMC10133699; doi:10.3389/fphys.2023.1108966)
Supplement: Supplementary file 3 [file Image1.PDF]

Supplementary Material

A novel OSA-related model of intermittent hypoxia in endothelial cells under flow reveals pronounced inflammatory pathway activation

Martin B. Müller*, Clemens Stihl, Annika Schmid, Simon Hirschberger, Rea Mitsigiorgi, Martin Holzer, Martin Patscheider, Bernhard G Weiss, Christoph Reichel, Max Hübner, and Bernd Uhl

*** Correspondence:** Corresponding Author: Dr. Martin B. Müller
Martin_Bernhard.Mueller@med.uni-muenchen.de

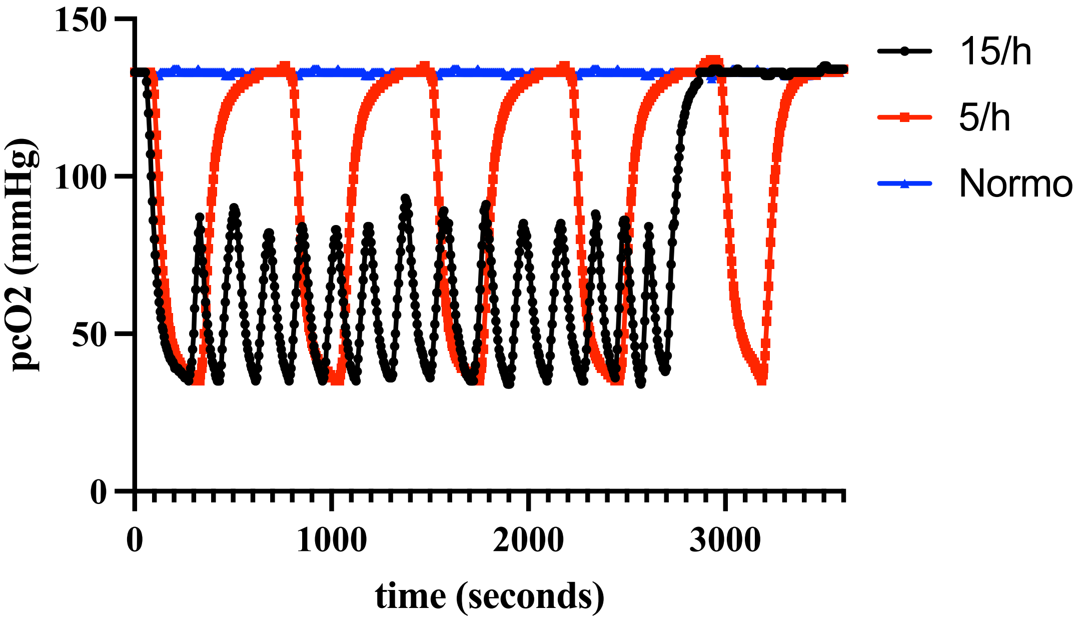

Supplementary Figure 1. Detailed partial pressure of oxygen on cellular level (pcO_2) over one hour measured by LiCox® probe in the normoxia (normo), 5 cycles/hour (5/h) and 15 cycles/hour (15/h) group. Shown are 15 subsequent hypoxia/reoxygenation cycles in the 15/h group and 5 cycles in the 5/h group.
